# A Model Study of the Photochemical Fate of As(III) in Paddy-Water

**DOI:** 10.3390/molecules22030445

**Published:** 2017-03-11

**Authors:** Luca Carena, Davide Vione

**Affiliations:** 1 Dipartimento di Chimica, Università di Torino, Via Pietro Giuria 5, 10125 Torino, Italy; luca.carena@unito.it; 2 Centro Interdipartimentale NatRisk, Università di Torino, Largo Paolo Braccini 2, 10095 Grugliasco (TO), Italy

**Keywords:** arsenic contamination, paddy-field floodwater, sunlight-induced reactions

## Abstract

The APEX (Aqueous Photochemistry of Environmentally-occurring Xenobiotics) software previously developed by one of us was used to model the photochemistry of As(III) in paddy-field water, allowing a comparison with biotic processes. The model included key paddy-water variables, such as the shielding effect of the rice canopy on incident sunlight and its monthly variations, water pH, and the photochemical parameters of the chromophoric dissolved organic matter (CDOM) occurring in paddy fields. The half-life times (*t*_1/2_) of As(III) photooxidation to As(V) would be ~20–30 days in May. In contrast, the photochemical oxidation of As(III) would be much slower in June and July due to rice-canopy shading of radiation because of plant growth, despite higher sunlight irradiance. At pH < 8 the photooxidation of As(III) would mainly be accounted for by reaction with transient species produced by irradiated CDOM (here represented by the excited triplet states ^3^CDOM^*^, neglecting the possibly more important reactions with poorly known species such as the phenoxy radicals) and, to a lesser extent, with the hydroxyl radicals (HO^•^). However, the carbonate radicals (CO_3_^•−^) could be key photooxidants at pH > 8.5 provided that the paddy-water ^3^CDOM^*^ is sufficiently reactive toward the oxidation of CO_3_^2−^. In particular, if paddy-water ^3^CDOM^*^ oxidizes the carbonate anion with a second-order reaction rate constant near (or higher than) 10^6^ M^−1^·s^−1^, the photooxidation of As(III) could be quite fast at pH > 8.5. Such pH conditions can be produced by elevated photosynthetic activity that consumes dissolved CO_2_.

## 1. Introduction

Arsenic (As) contamination of paddy fields is an important pollution problem in several regions around the world, such as the Bengal Basin area [1]. Contamination by As is usually caused by irrigation of paddy fields with groundwater containing elevated As levels [1,2]. In a paddy field, As can be found both in the soil and in the flooding water, and its concentration range can be quite wide [3]. Moreover, several types of rice plants are able to uptake soil As in the roots and to accumulate it, leading to human exposure through food [3,4,5]. There are many processes governing As chemistry in paddy fields, which depend on the physicochemical and biological features of paddies and may affect As speciation. The main As redox states are As(V) and As(III), usually occurring as inorganic compounds (arsenate and arsenite, respectively) and, to a minor extent, as organic species, such as the methylated As forms [6]. Under aerobic soil conditions that occur before the flooding of rice crops, As is sorbed as As(V) because the oxyanion arsenate has high affinity for soil minerals and, particularly, for Fe (hydr)oxides. Anaerobic regimes develop into the paddy soil after flooding, and Fe(III) (hydr)oxides and As(V) undergo fast microbial reduction. Arsenic is, thus, released as As(III) into the soil pore water, because the more mobile (and toxic) As(III) has lesser affinity than As(V) towards soil minerals [7]. The results reported by Takahashi et al. [7] suggest that the half-life time (*t*_1/2_) of As(V) is ~30 days. The produced aqueous As(III) could subsequently undergo oxidation to As(V) in the upper soil layers, where oxygen occurs in higher amounts. In particular, biotic oxidation takes place into the oxygenated bulk soil and in the rhizosphere of the rice plant roots, where Fe (hydr)oxides form an iron plaque and As is re-sorbed as As(V) [2,8]. The biotic oxidation of As(III) occurs mainly as a detoxification process, but As(III) can also be used as an electron/energy source during the growth of microorganisms. Biotic detoxification of As(III) to As(V) may be fast, and apparent *t*_1/2_ values have been found to be <1 day depending on experimental conditions (although the fastest processes might not always be representative of actual paddies) [9]. As(V) can, in turn, undergo biotic reduction to As(III) in both aerobic and anaerobic environments, by dissimilatory arsenate-reduction processes [10], which may be fast under anaerobic conditions [11]. As(V) reduction is also fast in aerobic environments, and the *t*_1/2_ of As(V) has been found to be <3 days [9,12]. Therefore, microbial oxidation/reduction processes are key phenomena of As speciation in paddy soil and in surface water environments [13]. They could also be important in the paddies floodwater, where light is an important factor as well.

Photochemistry plays a key role in the transformation of inorganic and organic substances in surface water. The photochemical reactions occurring in sunlit natural waters can generally be divided into direct and indirect phototransformation pathways. The direct photolysis of a substrate takes place after absorption of sunlight, if the absorption spectrum of the substance overlaps with the sunlight spectrum. In contrast, indirect photochemistry is triggered by compounds called photosensitizers, which occur naturally in surface waters and are able to absorb sunlight. The main photosensitizers are the chromophoric moieties of dissolved organic matter (chromophoric dissolved organic matter or CDOM), nitrate and nitrite. The irradiated photosensitizers produce high-energy transient species, such as hydroxyl radicals (HO^•^), carbonate radicals (CO_3_^•−^), singlet oxygen (^1^O_2_), and the excited triplet states of CDOM (^3^CDOM^*^). These transient species react with dissolved substrates and cause their indirect phototransformation [14]. As(III) is well known to react with both HO^•^ and ^3^CDOM^*^ [15,16,17,18], whereas its reactivity with carbonate radicals has been studied only under alkaline conditions [15]. As(III) reactivity toward ^1^O_2_ was found to be negligible in solutions containing humic substances under irradiation [18]. Direct As(III) photolysis could occur significantly at λ = 254 nm [19], but it is not an important photochemical pathway under sunlight [16,17]. 

It should be pointed out that the oxidation of As(III) in the presence of irradiated CDOM likely involves radical species (presumably phenoxy radicals) to a higher extent than ^3^CDOM^*^ [18]. Unfortunately, little to nothing is known about the formation and reactivity of these radicals, which prevents photochemical modelling. In the present study, the photochemical fate of As(III) in rice-field water was modelled using the available literature values of the second-order rate constants of the reactions between As(III) and several transients (^•^OH, ^3^CDOM^*^, CO_3_^•−^), as well as the photochemical parameters of paddy floodwater. This approach provides a lower limit for the photochemical oxidation kinetics of As(III), by neglecting the reactions involving the phenoxy radicals.

## 2. Results and Discussion

**Figure 1:**
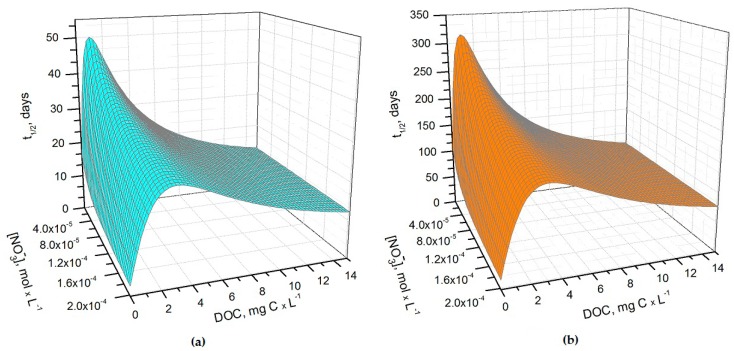
(**a**) Photochemical half-life time (*t*_1/2_) of H_3_AsO_3_ in rice field water in May. Water conditions: 5 cm depth; 1 μM NO_2_^−^, 1 mM bicarbonate, 10 μM carbonate, and 0.53 sunlight transmittance; (**b**) Photochemical half-life time of H_3_AsO_3_ in rice field water in June. Water conditions: 5 cm depth; 1 μM NO_2_^−^, 1 mM bicarbonate, 10 μM carbonate and 0.08 sunlight transmittance. The day units of *t*_1/2_ are referred to average mid-latitude irradiance conditions occurring in mid-May and mid-June, respectively.

The half-life time (*t*_1/2_) of As(III), in the form of H_3_AsO_3_, in sunlit rice field water was modelled for the months of May, June, and July. This period follows the flooding of paddies in late April–early May. The details of the photochemical model are reported in the Methods section. Figure 1a,b show the half-life times of H_3_AsO_3_ in May and June as a function of nitrate concentration and of the dissolved organic carbon (DOC) content of paddy water. The light transmittance through the rice plant canopy was taken into account in the model, considering that the canopy shading effect increases as the rice plants grow higher. Consequently, during the rice growing season there can be considerable variations in the sunlight irradiance that reaches the paddy-water surface [20]. Sunlight transmittance through the rice canopy has been measured in the La Albufera coastal lagoon (Spain, latitude ~39°N) during the months of May, June, and July, 1989 [21]. The reported monthly values of the average transmittance were ~53%, ~8% and ~6%, respectively. These values were used to model As(III) photochemistry in the present work.

**Figure 2:**
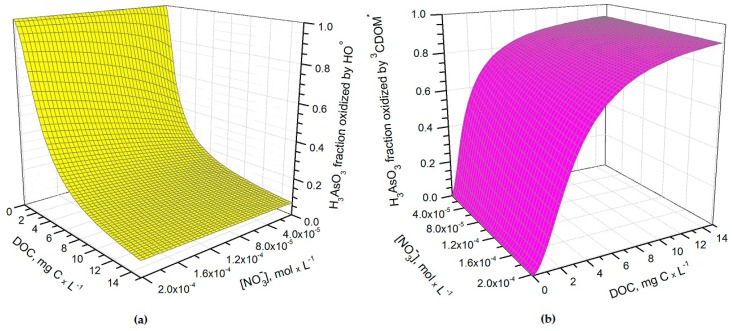
Fraction of H_3_AsO_3_ transformed by (**a**) hydroxyl radicals and (**b**) CDOM triplet states (^3^CDOM*) in May, as a function of nitrate and dissolved organic carbon (DOC). Water conditions: 5 cm depth, 1 μM NO_2_^−^, 1 mM bicarbonate, and 10 μM carbonate. Note that direct photolysis, carbonate radicals and singlet oxygen reactions were not taken into account in the model.

It can be seen from Figure 1 that *t*_1/2_ undergoes important variations with nitrate concentration and DOC. In particular, at low DOC values (< 1 mg·C·L^−1^) the increase of nitrate causes a significant *t*_1/2_ decrement, because in such conditions HO^•^ that is partially photogenerated by nitrate plays a major role in As(III) photochemical oxidation (see also Figure 2a, which reports the fraction of H_3_AsO_3_ oxidized by HO^•^ as a function of DOC and nitrate). Low-DOC conditions are scarcely representative of the rice field floodwater environment, however, because a significant amount of dissolved organic matter (DOM, not necessarily chromophoric) is released by soil/sediment and by the rice plants. When the DOC is higher (>6 mg·C·L^−1^, more representative of paddy floodwater), nitrate has a limited influence on the rate of As(III) photooxidation. Under these circumstances CDOM is the main chromophore, it limits the sunlight absorption by nitrate and it is also the main photochemical HO^•^ source. Moreover, DOM (note that high-DOC waters are DOM-rich by definition [22]) is a major HO^•^ scavenger and causes the steady-state [HO^•^] to be low. In contrast, [3CDOM*] is higher at high DOC [14] and, in these circumstances, the triplet-sensitised processes can play an important role in As(III) photooxidation (see Figure 2b). The maximum values of *t*_1/2_ occur at DOC = 1–2 mg·C·L^−1^ (Figure 1). In these conditions, there is already enough DOM to scavenge HO^•^ significantly, but not yet enough CDOM to produce elevated [3CDOM*]. 

**Figure 3:**
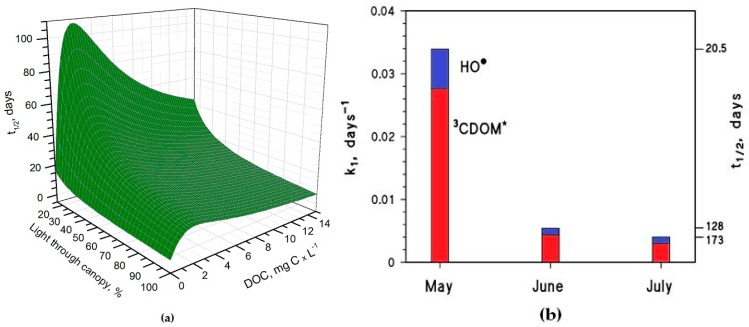
(**a**) Photochemical half-life time of H_3_AsO_3_ in rice-field water, as a function of the DOC and of light transmittance through the rice canopy. Other conditions: 5 cm depth, 0.1 mM NO_3_^−^, 1 μM NO_2_^−^, 1 mM bicarbonate, 10 μM carbonate, and May sunlight. (**b**) Pseudo-first-order rate constant of H_3_AsO_3_ photooxidation in the period from May to July. The corresponding half-life times (*t*_1/2_ = ln2 (*k*_1_)^−^^1^) are also reported in the right Y-axis. Water conditions: 5 cm depth, 7 mg·C·L^−1^ DOC, 0.1 mM NO_3_^−^, 1 μM NO_2_^−^, 1 mM bicarbonate, and 10 μM carbonate. Transmittance values through the rice canopy are reported in the main text. The days are referred to average irradiance conditions occurring at mid latitude in, where relevant, mid-May, mid-June, and mid-July.

It is interesting to note that the t_1/2_ values are lower in May than in June, despite the significantly higher sunlight irradiance in June [14]. The reason is that the rice plants are higher in June, and their canopy produces a considerable decrease of the irradiance over the water surface. The shading by the rice canopy deeply affects all the photochemical processes as shown in Figure 3a, which reports the *t*_1/2_ of As(III) as a function of water DOC and canopy transmittance. Figure 3b shows the overall As(III) photooxidation kinetics in May through July, which is affected both by sunlight irradiance and by shading from the rice canopy. It is clear that photooxidation is faster in May, while limited differences can be observed between June and July. Photochemical processes are predicted to be quite slow in June and July due to canopy shading. In contrast, the value t_1/2_ ~ 20 days in May is, interestingly, of the same order of magnitude as the As(III) level fluctuations observed in a laboratory experiment that used paddy water and soil [7]. As(III) photooxidation may, thus, potentially play a role in As processing in paddy fields in the month of May, but in definite circumstances it has been found that the As redox processes in paddy soil and in the rice rhizosphere may be considerably faster [8,9,12].

Under DOC conditions that are typically representative of paddy water, such as those assumed in the modelling of Figure 3b, ^3^CDOM^*^ would be the main photooxidant for As(III) and account for ~80% of its transformation, while the remaining ~20% could be ascribed to HO^•^. This finding is consistent with the experimental results reported by Buschmann et al. in irradiation experiments of As(III) solutions containing humic substances [18], where the role of HO^•^ in As(III) phototransformation was a minor one. Note that the relative importance of ^3^CDOM* and HO^•^ in As(III) photooxidation does not depend on the light transmittance through the rice canopy, but it is rather a function of the water chemistry. Moreover, it should be recalled here that the reaction between As(III) and ^3^CDOM^*^ is only a lower limit for the reaction kinetics between As(III) and irradiated CDOM, because the main oxidative process is actually carried out by additional transients (possibly phenoxy radicals) [18].

The above discussion refers to photochemical scenarios in which HO^•^ and ^3^CDOM^*^ are assumed to be the main photooxidants of As(III). However, H_3_AsO_3_ is in equilibrium with its conjugate base As(OH)_2_O^−^ (pK_a_ = 9.2), for which the photoreactivity has been studied in basic conditions only [15]. To carry out a more complete description of As(III) indirect photochemistry, pH was included into the model (see Methods for the mathematical treatment). By considering pH as a master variable and introducing the photoreactivity of As(OH)_2_O^−^, the role of CO_3_^•−^-induced oxidation gains importance as a photochemical process. The radical CO_3_^•−^ is produced by bicarbonate and carbonate oxidation (Reactions 1–3), and the corresponding second-order reaction rate constants are $k_{HCO_{3}^{-}}^{HO^{•}}$ = 8.5·10^6^ M^−1^ s^−1^, $k_{CO_{3}^{2-}}^{HO^{•}}$ = 3.9·10^8^ M^−1^ s^−1^, as well as $k_{CO_{3}^{2-}}^{{}^{3}{CDOM}^{*}}$. In particular, we found that the reactivity of ^3^CDOM^*^ with the carbonate anions (expressed by the parameter $k_{CO_{3}^{2-}}^{{}^{3}{CDOM}^{*}}$, see Reaction 3) is a key variable of the model. The value of $k_{CO_{3}^{2-}}^{{}^{3}{CDOM}^{*}}$ has been found to vary widely depending on the proxy molecules (triplet sensitizers) used to study the reactivity between CO_3_^2−^ and ^3^CDOM^*^ [23].

(1) $${\mathrm{HO}}^{•}+{{\mathrm{HCO}}_{3}}^{-}\overset{k_{HCO_{3}^{-}}^{HO^{•}}}{\to }H_{2}O+{{\mathrm{CO}}_{3}}^{•-}$$

(2) $${\mathrm{HO}}^{•}+{{\mathrm{CO}}_{3}}^{2-}\overset{k_{CO_{3}^{2-}}^{HO^{•}}}{\to }{\mathrm{HO}}^{-}+{{\mathrm{CO}}_{3}}^{•-}$$

(3) $${}^{3}{\mathrm{CDOM}}^{*}+{{\mathrm{CO}}_{3}}^{2-}\overset{k_{CO_{3}^{2-}}^{{}^{3}{CDOM}^{*}}}{\to }{\mathrm{CDOM}}^{•-}+{{\mathrm{CO}}_{3}}^{•-}$$

**Figure 4:**
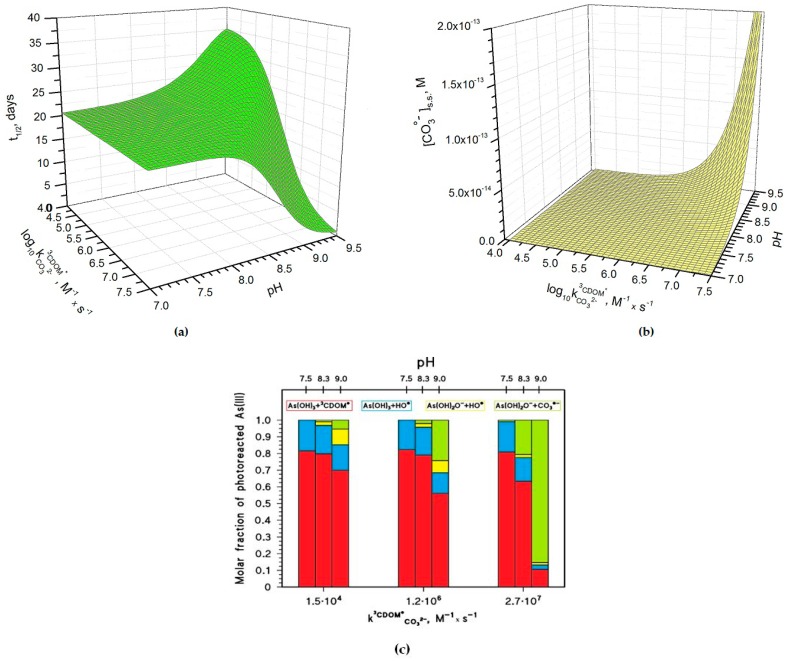
Photochemical parameters of As(III) photooxidation computed for the month of May. Water conditions: 5 cm depth, 0.1 mM NO_3_^−^, 1 μM NO_2_^−^, inorganic carbon (IC) = 1.1 mmol·C·L^−1^, and DOC = 7 mg·C·L^−1^. (**a**) Half-life time of As(III) and (**b**) steady-state concentrations of the carbonate radicals, as a function of pH and of the decimal logarithm of $k_{CO_{3}^{2-}}^{{}^{3}{CDOM}^{*}}$. The days are referred to average irradiance conditions occurring at mid-latitude in mid-May. (**c**) Roles of the main reactions accounting for the photooxidation of dissolved As(III). The $k_{CO_{3}^{2-}}^{{}^{3}{CDOM}^{*}}$ values used in (**c**) are those reported for the three CDOM proxy molecules 3’-methoxyacetophenone (1.5·10^4^ M^−1^·s^−1^), benzophenone (1.2·10^6^ M^−1^·s^−1^) and duroquinone (2.7·10^7^ M^−1^·s^−1^) [23].

Figure 4a shows how the pH and the $k_{CO_{3}^{2-}}^{{}^{3}{CDOM}^{*}}$ rate constant values affect the half-life time of As(III) in paddy floodwater in May. For pH < 8, $k_{CO_{3}^{2-}}^{{}^{3}{CDOM}^{*}}$ and pH have a scarce influence on As(III) photochemistry and one has *t*_1/2_ ~20 days, in analogy with the results reported in Figure 1 and Figure 3. In this scenario, As(III) photooxidation would be mainly accounted for by the reactions between H_3_AsO_3_ and ^3^CDOM^*^/HO^•^ (see also Figure 4c, which reports the fractions of As(III) photooxidation accounted for by HO^•^, CO_3_^•−^, and ^3^CDOM^*^, for different values of pH and of $k_{CO_{3}^{2-}}^{{}^{3}{CDOM}^{*}}$). Indeed, the molar fraction of As(OH)_2_O^−^ ($\alpha_{As{\left(OH\right)}_{2}O^{-}}$, see the Methods section) and the steady-state [CO_3_^•−^] (Figure 4b) are too low at pH < 8 for CO_3_^•−^ to be involved significantly in As(III) oxidation.

At pH > 8–8.5 one has different trends depending on the value of $k_{CO_{3}^{2-}}^{{}^{3}{CDOM}^{*}}$. If $k_{CO_{3}^{2-}}^{{}^{3}{CDOM}^{*}}$ is below 10^6^ M^−1^·s^−1^, *t*_1/2_ has some increase with increasing pH (Figure 4a), because [CO_3_^•−^] is too low in these conditions (see Figure 4b) to significantly affect the photooxidation of As(III), and because As(OH)_2_O^−^ is less reactive than H_3_AsO_3_ towards HO^•^ and ^3^CDOM^*^ [15,18]. In this case, the photooxidation of H_3_AsO_3_ prevails as the As(III) transformation process (see Figure 4c). In contrast, if $k_{CO_{3}^{2-}}^{{}^{3}{CDOM}^{*}}$ is around 10^6^ M^−1^·s^−1^ or higher, one has an important decrease of *t*_1/2_ with increasing pH. The main reason is the reaction between As(OH)_2_O^−^ and CO_3_^•−^ (see Figure 4c), which is enhanced by the parallel increase with pH of both $\alpha_{As{\left(OH\right)}_{2}O^{-}}$ and [CO_3_^•−^]. 

**Figure 5:**
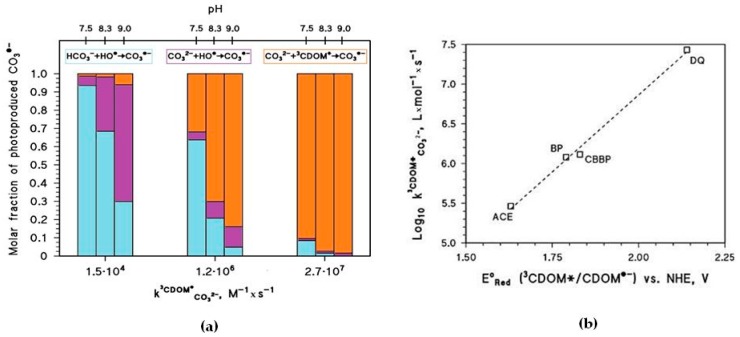
(**a**) Relative contributions of Reactions 1–3 to the production of CO_3_^•−^ in the scenario described by Figure 4. (**b**) Correlation plot between the reduction potentials of some excited triplet states and their reactivity toward the carbonate anion, expressed as second-order reaction rate constants: ACE = acetophenone; BP = benzophenone; CBBP = 4-carboxybenzophenone; DQ = duroquinone. The $k_{CO_{3}^{2-}}^{{}^{3}{CDOM}^{*}}$ values used are those reported in [23].

The pH increase of [CO_3_^•−^] shown in Figure 4b is mainly due to the reaction between CO_3_^2−^ and ^3^CDOM^*^, and it is expected to occur only if $k_{CO_{3}^{2-}}^{{}^{3}{CDOM}^{*}}$ ~ 10^6^ M^−1^·s^−1^ or higher. The important role of $k_{CO_{3}^{2-}}^{{}^{3}{CDOM}^{*}}$ is additionally highlighted in Figure 5a, which reports the relative weight of the various CO_3_^•−^ generation processes for different values of pH and $k_{CO_{3}^{2-}}^{{}^{3}{CDOM}^{*}}$. The value of $k_{CO_{3}^{2-}}^{{}^{3}{CDOM}^{*}}$ has a clear correlation with the triplet-state reduction potential, as shown in Figure 5b. In the presence of triplet states with sufficiently high oxidizing power (e.g., E°(^3^CDOM^*^/CDOM^•−^) > 1.7 V, causing significant CO_3_^•−^ production) and at basic pH, *t*_1/2_ could reach very low values (below one week) and make photochemistry a very significant factor in the As redox cycling.

In the presence of sufficiently reactive ^3^CDOM^*^, favourable pH conditions for As(III) photooxidation could be produced by algal photosynthesis that consumes dissolved CO_2_ and may considerably increase the water pH. The reaction rate constants between paddy-field ^3^CDOM^*^ and CO_3_^2−^ are presently unknown and the topic deserves further investigation, but the occurrence of humic substances in paddy water [24] may suggest a non-negligible photoreactivity of the relevant CDOM [25].

## 3. Methods (Photochemical Modelling)

The assessment of As(III) photooxidation was carried out with the APEX Aqueous Photochemistry of Environmentally-occurring Xenobiotics) software, which is freely available as electronic supplementary information of [26]. APEX allows for the modelling of the photochemistry of surface-water environments (in the form of steady-state concentrations of photogenerated transients) and of the photochemical fate of xenobiotics. APEX computes the xenobiotics pseudo-first-order transformation rate constants (*k*_1_) and half-life times (*t*_1/2_ = ln2 (*k*_1_)^−1^) [26] as a function of sunlight irradiance, water (photo)chemistry, and depth. For this purpose, APEX has been used successfully to investigate the photochemical behaviour of several xenobiotics and the formation of their phototransformation intermediates [27,28,29,30]. APEX requires input data concerning water depth, chemical composition (nitrate, nitrite, dissolved organic carbon, and inorganic carbon species) and photochemistry (absorption spectrum and formation quantum yields of transient species by CDOM). Moreover, second-order reaction rate constants with the photogenerated transients and photolysis quantum yields are key parameters to model the photochemical fate of xenobiotics. 

APEX uses as standard sunlight irradiance and standard sunlight spectrum the quantities relative to fair-weather 15 July at mid latitude (45° N), at 9 a.m. or 3 p.m. solar time. It also allows an approximate assessment of photochemistry throughout the year thanks to the *APEX_season* function, which operates corrections in order to compute transient steady-state concentrations and photoreaction kinetics in different months. However, APEX does not automatically take into account the effect of sunlight shielding by the rice plant canopy. The photochemistry of paddy floodwater occurs during the rice-growing season from May to July, and the shielding effect was included in the model by considering the transmittance (*T*) of sunlight through the plant canopy in each month (i.e., literature-available $T^{month}$, where month is May, June or July). Be $k_{1}^{month}$ the APEX-modelled photooxidation kinetics (first-order decay constant) of As(III) without taking into account the shielding effect. The correction was carried out by computing $(k_{1}^{month})\prime =k_{1}^{month}\times T^{month}$. It must be pointed out that $T^{month}$ was considered to be wavelength-independent, which is justified by the fact that the size of the shielding medium (plant leaves and stems) is much longer than the light wavelengths. Another key input parameter in the model is the water depth, *d*. Depth, together with the solar zenith angle (*z*), influences the optical path length of sunlight into the water. Here, a value of 5 cm was assumed for the water depth, which is typical of paddy fields in May and undergoes some non-substantial increase in the following months. By considering a constant water depth one slightly overestimates the photochemistry in June and July, but calculations showed that photochemical reactions would, in any case, be negligible in these months due to light shielding by the rice canopy. The light optical path length *l* was obtained as $l=d\;{\left[\sqrt{(1-{\left(n^{-1}\times \sin z\right)}^{2}}\right]}^{-1}$, where *n* = 1.34 is the refraction index of water and *z* is the zenith angle. As a result, *l* > *d* was 6 cm in May and 5.7 cm in June and July [26].

The chemical and photochemical parameters of paddy water have been set in analogy with the results of previous work [24], carried out on paddy-water samples from three rice farms located in the province of Vercelli (Piedmont region, northwestern Italy). In particular, the average formation quantum yields of the transient species *i* from irradiated CDOM ($\Phi_{i}^{CDOM}$) had the following values: $\Phi_{{HO}^{•}}^{CDOM}$ = 1.7·10^−5^; $\Phi_{{}^{3}{CDOM}^{*}}^{CDOM}$ = 2.9·10^−2^, and $\Phi_{{}^{1}O_{2}}^{CDOM}$ = 9·10^−3^. The chemical analysis of the paddy water samples yielded on average DOC ~7 mg·C·L^−1^ and inorganic carbon (IC) ~13 mg·C·L^−1^ (namely, ~10^−3^ mol·C·L^−1^). Nitrate and nitrite were under the limit of detection in two out of three samples, the third one having 1.7 mg·N·L^−1^ nitrate and 18 μg·N·L^−1^ nitrite (namely, 1.2 mmol·L^−1^ nitrate and 1.3 µmol·L^−1^ nitrite). Similar values of nitrate concentration have been reported for flooded paddy fields in Spain [21]. 

The modelling of As(III) photochemistry was initially carried out by considering the reactions of H_3_AsO_3_ with HO^•^ and ^3^CDOM^*^, for which the following second-order rate constants were used: $k_{H_{3}AsO_{3}}^{HO^{•}}$ = 8.5·10^9^ M^−1^·s^−1^ [15,16], and $k_{H_{3}AsO_{3}}^{{}^{3}{CDOM}^{*}}$ = 1.6·10^7^ M^−1^·s^−1^ [18]. The reaction of As(III) with HO^•^ forms an As(IV) species that is quite unstable under aerated conditions and is quickly oxidized to As(V) upon reaction with dissolved oxygen, following different possible pathways [15,16]. Since paddy water is oxygen-rich during the day due to photosynthesis [31,32], we considered the reaction between As(IV) and O_2_ to be very fast and assumed the oxidation of As(III) by HO^•^ to be the rate-determining step of As(V) production. 

The reactivity of As(III) with CO_3_^•−^ has been investigated in the literature only in basic conditions (pH > 9), and the second-order rate constant has been reported as $k_{As{\left(OH\right)}_{2}O^{-}}^{CO_{3}^{•-}}$ = 1.1·10^8^ M^−1^·s^−1^ [15]. Since H_3_AsO_3_ has pK_a_ = 9.2, the reaction between CO_3_^•−^ and As(OH)_2_O^−^ should be important only at basic pH where As(OH)_2_O^−^ is a significant As(III) species. Singlet oxygen has been found to react slowly with As(III) [18], and it was thus neglected in our model. It must be pointed out that phenoxy radicals, produced by CDOM irradiation, could be key photooxidants of As(III) [18]. Unfortunately, these reactive species are very difficult to take into account in a photochemical model because of insufficient knowledge concerning the amount of phenolic domains in DOM and their possible variability in different environments. Moreover, the formation quantum yields of phenoxy radicals and their second-order rate constants with xenobiotics are not known. For this reason, the present photochemical model did not take into account the oxidation of As(III) by phenoxy radicals. Therefore, the photochemistry of As(III) in rice field water might be underestimated.

The photochemical pseudo-first order rate constants of As(III) oxidation were also modelled as a function of water pH, by assuming that the total concentration of dissolved As(III) (namely, [As(III)]_tot_) is the sum of [H_3_AsO_3_] and [As(OH)_2_O^−^]. Each concentration can be expressed as a function of [As(III)]_tot_ and pH, by using the molar fractions α_x_ (where *x* = H_3_AsO_3_ or As(OH)_2_O^−^):

(4) $$[H_{3}AsO_{3}]={\left[As(III)\right]}_{tot}\;\alpha_{H_{3}AsO_{3}}={\left[As(III)\right]}_{tot}\;\frac{\left[H^{+}\right]}{[H^{+}]+K_{a}}$$

(5) $$[As{\left(OH\right)}_{2}O^{-}]={\left[As(III)\right]}_{tot}\;\alpha_{As{\left(OH\right)}_{2}O^{-}}={\left[As(III)\right]}_{tot}\;\frac{K_{a}}{[H^{+}]+K_{a}}$$

The photochemical reactions taken into account are listed below:

(6) $$H_{3}{\mathrm{AsO}}_{3}\overset{HO^{•}}{\to }\mathrm{As}(\mathrm{IV})\overset{fast}{\to }\mathrm{As}(V)$$

H_3_AsO_3_ + ^3^CDOM^*^ → As(V) (7)

(8) $$\mathrm{As}{\left(\mathrm{OH}\right)}_{2}O^{-}\overset{HO^{•}}{\to }\mathrm{As}(\mathrm{IV})\overset{fast}{\to }\mathrm{As}(V)$$

(9) $$\mathrm{As}{\left(\mathrm{OH}\right)}_{2}O^{-}\overset{CO_{3}^{•-}}{\to }\mathrm{As}(\mathrm{IV})\overset{fast}{\to }\mathrm{As}(V)$$

Most of the relevant second-order reaction rate constants are available in the literature but, because the rate constant of As(OH)_2_O^−^ + HO^•^ is not known, we assumed it to be equal to the corresponding H_3_AsO_3_ rate constant. This assumption is supported by the fact that, according to Kim et al. [16], the phototransformation rate of As(III) by HO^•^ in the 4.5–12 pH interval is mostly affected by [HO^•^] variations and much less by the speciation of As(III). The total phototransformation rate of As(III) ($R_{As(III)}^{tot}$) can be written as the sum of the separate rates of Reactions (6–9), as follows:

(10) $$R_{As(III)}^{tot}=R_{H_{3}AsO_{3}}^{HO^{•}}+R_{H_{3}AsO_{3}}^{{}^{3}{CDOM}^{*}}+R_{As{\left(OH\right)}_{2}O^{-}}^{HO^{•}}+R_{As{\left(OH\right)}_{2}O^{-}}^{CO_{3}^{•-}}$$

where $R_{As(III)}^{i}=k_{As(III)}^{i}\;[As(III)][i]$, *i* = HO^•^, ^3^CDOM* or CO_3_^•−^, and As(III) = H_3_AsO_3_ or As(OH)_2_O^−^. By introducing Equations (4) and (5) in Equation (10) and by dividing for [As(III)]_tot_, one obtains:

(11) $$k_{As(III)}^{tot}=\alpha_{H_{3}AsO_{3}}(k_{H_{3}AsO_{3}}^{HO^{•}}\;[HO^{•}]+k_{H_{3}AsO_{3}}^{{}^{3}{CDOM}^{*}}\;[{}^{3}{CDOM}^{*}])+\alpha_{As{\left(OH\right)}_{2}O^{-}}(k_{As{\left(OH\right)}_{2}O^{-}}^{HO^{•}}\;[HO^{•}]+k_{As{\left(OH\right)}_{2}O^{-}}^{CO_{3}^{•-}}\;[CO_{3}^{•-}])$$

where $k_{As(III)}^{tot}=R_{As(III)}^{tot}{\left({\left[As(III)\right]}_{tot}\right)}^{-1}$ is the overall pseudo-first order rate constant of As(III) oxidation. Since $k_{As(III)}^{tot}$ contains the terms $\alpha_{H_{3}AsO_{3}}$ and $\alpha_{As{\left(OH\right)}_{2}O^{-}}$, it is a function of pH as suggested by Equations (4) and (5). 

The steady-state [CO_3_^•−^] obviously depends on the value of $k_{CO_{3}^{2-}}^{{}^{3}{CDOM}^{*}}$, for which different estimates are available [23]. The take the different possibilities into account, [CO_3_^•−^] was calculated as follows:

(12) $$[CO_{3}^{•-}]=\frac{\left[HO^{•}]\;(k_{HCO_{3}^{-}}^{HO^{•}}\;[HCO_{3}^{-}]+k_{CO_{3}^{2-}}^{HO^{•}}\;[CO_{3}^{2-}])+k_{CO_{3}^{2-}}^{{}^{3}{CDOM}^{*}}\;[{}^{3}{CDOM}^{*}]\;[CO_{3}^{2-}\right]}{k_{DOM}^{CO_{3}^{•-}}\;DOC}$$

where [HO^•^] and [3CDOM*] were provided by APEX, $k_{HCO_{3}^{-}}^{HO^{•}}$ = 8.5 × 10^6^ M^−1^·s^−1^, and $k_{CO_{3}^{2-}}^{HO^{•}}$ = 3.9 × 10^8^ M^−1^·s^−1^ [33]. Moreover, $k_{DOM}^{CO_{3}^{•-}}$ = 10^2^ L·(mg·C) ^−1^·s^−1^ is the second-order rate constant of CO_3_^•−^ scavenging by DOM.

## 4. Conclusions

Photochemical modelling suggests that As(III) would undergo photooxidation in paddy floodwater and, at pH < 8.5, mainly upon reaction with ^3^CDOM^*^ and HO^•^. Note that As(III) oxidation by irradiated CDOM likely involves phenoxy radicals or other species to a higher extent than ^3^CDOM^*^; thus, our approach can underestimate the importance of photochemical reactions. The predicted half-life times *t*_1/2_ were much lower in May compared to June and July because, in the latter months, the rice plant growth would produce an important sunlight-shielding effect by the canopy. Therefore, As(III) photooxidation can be neglected in June and July. When typical chemical composition data of paddy water in May are taken into account, one obtains As(III) half-life times in the range of 20–30 days. In this case, photochemical As(III) oxidation could be overcome by microbial As(V) reduction to As(III). However, the photooxidation of As(III) might be much faster at pH > 8.5 if the carbonate radicals, CO_3_^•−^, were produced efficiently by the reaction between ^3^CDOM^*^ and CO_3_^2−^. Much depends on the $k_{CO_{3}^{2-}}^{{}^{3}{CDOM}^{*}}$ second-order rate constant: if it is around 10^6^ M^−1^·s^−1^ or higher, the production of CO_3_^•−^ and the subsequent reaction between CO_3_^•−^ and As(OH)_2_O^−^ can play a key role in As(III) photooxidation. Further work is needed to better understand the CO_3_^•−^ formation processes in paddy water concerning, most notably, the CO_3_^•−^ formation quantum yield at basic pH and the $k_{CO_{3}^{2-}}^{{}^{3}{CDOM}^{*}}$ rate constant.
